# Physical Interactions With Bacteria and Protozoan Parasites Establish the Scavenger Receptor SSC4D as a Broad-Spectrum Pattern Recognition Receptor

**DOI:** 10.3389/fimmu.2021.760770

**Published:** 2021-12-24

**Authors:** Marcos S. Cardoso, Rita F. Santos, Sarah Almeida, Mónica Sá, Begoña Pérez-Cabezas, Liliana Oliveira, Joana Tavares, Alexandre M. Carmo

**Affiliations:** ^1^ Instituto de Investigação e Inovação em Saúde, Universidade do Porto, Porto, Portugal; ^2^ IBMC–Instituto de Biologia Molecular e Celular, Porto, Portugal; ^3^ Programa Doutoral em Biologia Molecular e Celular (MCbiology), Instituto de Ciências Biomédicas Abel Salazar, Universidade do Porto, Porto, Portugal; ^4^ Departamento de Biologia, Universidade de Aveiro, Aveiro, Portugal; ^5^ Doutoramento em Ciências Farmacêuticas (especialidade Microbiologia), Faculdade de Farmácia, Universidade do Porto, Porto, Portugal

**Keywords:** scavenger receptor cysteine-rich, pattern recognition receptors, bacteria, parasites, circulating receptors

## Abstract

Since the pioneering discoveries, by the Nobel laureates Jules Hoffmann and Bruce Beutler, that Toll and Toll-like receptors can sense pathogenic microorganisms and initiate, in vertebrates and invertebrates, innate immune responses against microbial infections, many other families of pattern recognition receptors (PRRs) have been described. One of such receptor clusters is composed by, if not all, at least several members of the scavenger receptor cysteine-rich (SRCR) superfamily. Many SRCR proteins are plasma membrane receptors of immune cells; however, a small subset consists of secreted receptors that are therefore in circulation. We here describe the first characterization of biological and functional roles of the circulating human protein SSC4D, one of the least scrutinized members of the family. Within leukocyte populations, SSC4D was found to be expressed by monocytes/macrophages, neutrophils, and B cells, but its production was particularly evident in epithelial cells of several organs and tissues, namely, in the kidney, thyroid, lung, placenta, intestinal tract, and liver. Similar to other SRCR proteins, SSC4D shows the capacity of physically binding to different species of bacteria, and this opsonization can increase the phagocytic capacity of monocytes. Importantly, we have uncovered the capacity of SSC4D of binding to several protozoan parasites, a singular feature seldom described for PRRs in general and here demonstrated for the first time for an SRCR family member. Overall, our study is pioneer in assigning a PRR role to SSC4D.

## Introduction

The initial sensing of an invasive pathogen is one of the most critical events during an infection and is mediated by germline-encoded pattern recognition receptors (PRRs), which identify and bind conserved pathogen-associated molecular patterns (PAMPs) of microbes. Many different families of PRRs displaying either target-specific or broad recognition of different types of microbes have been described. Membrane-bound Toll-like receptors (TLRs) or C-type lectin receptors bind or sense microbe-exposed PAMPs and initiate signaling cascades to trigger innate immune cell activation, whereas intracellular pathogens or their by-products are recognized by intracellular PRRs such as cytoplasmic NOD-like receptors or by RIG-I-like receptors and endosomal TLRs that identify microbial genetic material (1–4).

Recent work has revealed that pattern recognition is a common feature of many scavenger receptor cysteine-rich (SRCR) proteins. The macrophage scavenger receptor type I (MSR1) and MARCO plasma membrane trimeric proteins have long been known to bind bacteria or bacterial endotoxins and to promote microbial phagocytosis (5, 6), but only more recently it was described that the cell surface receptors CD6 and CD163 of T cells and macrophages, respectively, can recognize Gram-positive and Gram-negative bacteria (7, 8). By contrast, CD5 has not been shown to bind bacteria, but its extracellular domain interacts with fungal cell wall components (9).

A small subset of SRCR consists of secreted receptors that are therefore in circulation and as such they have exceptional features to intercept, recognize, and neutralize invasive microbes and thus to contain infections. Galectin-3-binding protein (MAC2BP, LGALS3BP) is a small mosaic protein that contains, besides an SRCR domain, a BTB dimerization domain and a BACK domain (10). Although historically viewed as a malignant tumor-associated antigen, this protein has recently been identified as a possible biomarker for human sepsis (11). Better known for their infection-related immune functions, the circulating proteins CD5 antigen-like (CD5L), also known as apoptosis inhibitor expressed by macrophages (AIM) or secreted protein α (Spα) (12, 13), soluble scavenger protein with 5 SRCR domains (SSC5D) (14), and deleted in malignant brain tumors 1 (DMBT1) (15), containing respectively three, five, and 14 SRCR domains, display characteristic PRR features including a strong avidity to bind Gram-positive and Gram-negative bacteria (16–18).

Compared with the wealth of information gathered on the various roles of, for example, CD5L, spreading across a multitude of functions in numerous biological systems and phenomena (19), the attention on the very similar SSC4D protein has been almost inexistent. SSC4D is a 575-amino acid (aa)-long protein containing an N-terminal signal peptide, no transmembrane-encoding sequence, and four SRCR domains, all indicating that SSC4D is the last member of the subgroup of circulating SRCR proteins (20). In fact, SSC4D can be found in human blood plasma, albeit at a very low concentration (1 ng/ml) (21, 22). Although no extensive protein characterization, tissue distribution, or functional studies have been performed, northern blotting analyses imply that SSC4D is well expressed in the human kidney and placenta and moderately expressed in the liver, small intestine, spleen, and thymus (20).

Here, we describe the first comprehensive data on the roles and distribution of the SSC4D glycoprotein in a mammalian organism and how the evidence obtained clearly indicates that SSC4D functionally belongs to the PRR arm within the SRCR family.

## Materials and Methods

### Recombinant Scavenger Receptor Cysteine-Rich Proteins

Recombinant soluble proteins were produced in human embryonic kidney 293T cells and supplied in lyophilized form by INVIGATE GmbH. Specifically, recombinant forms of human CD5L and of the extracellular domain of human CD6 were produced from templates already described (17, 23) and modified to obtain chimeric proteins containing a signal peptide, the specific CD5L (Ser^20^ to Gly^347^) or CD6 (Asp^25^ to Glu^398^) sequences, HA and BirA recognition sequences, and 8—12-His tag sequences. Recombinant human SSC4D (UniProtKB accession no. Q8WTU2) was produced in a similar manner to include the specific protein sequence spanning domains 1–4 (Leu^48^-Ser^575^) of SSC4D. Recombinant human SSC4D-d1d2 (spanning SRCR domains 1 and 2) and SSC4D-d3d4 (domains 3 and 4) were produced to result in the SSC4D sequences Leu^48^-Gly^318^ and Ser^324^-Ser^575^ being fused to 8·His tag sequences.

For the expression in Caco-2 cells of full-length SSC4D fused to citrine and containing an HA tag, cDNA was amplified by PCR from Hep G2 cells using forward (5′-TAGACGCGTATGCACAAGGAAGCAGAGA-3′) and reverse (5′-CTAGGATCCCGAGCGTAGTCTGGGACGTCGTATGGGTATGAAGGCTGGCACAGGACACT-3′) primers. The resulting PCR product was cloned into the lentiviral expression vector pHR-mCitrine, using *Mlu*I and *Bam*HI restriction sites, to be under the control of the spleen focus-forming virus (SFFV) promoter and transduced into Caco-2 cells.

### Analysis of SSC4D Protein Expression

Cell lysates were prepared using radioimmunoprecipitation assay (RIPA) lysis buffer containing a mixture of protease and phosphatase inhibitors (Sigma-Aldrich). Protein concentration was measured by Bradford assay (Bio-Rad), and 60 μg of each sample were denatured in Laemmli’s sample buffer at 95°C for 10 min. Cell lysates and supernatants were separated by sodium dodecyl sulfate polyacrylamide gel electrophoresis (SDS-PAGE) and transferred to nitrocellulose membranes using Trans-Blot Turbo Transfer System (Bio-Rad). Membranes were blocked with 5% non-fat dried milk in Tris-buffered saline, 0.1% Tween 20 (TBS-T) for 1 h and probed with rabbit anti-SSC4D polyclonal antibody (raised against polypeptides corresponding to sequences R346-C364 and E470-R485 of mouse SSC4D; BIOTEM), followed by a goat anti-rabbit horseradish peroxidase (HRP) secondary antibody (Sigma-Aldrich). Immunoblots were developed using enhanced chemiluminescence (ECL) detection reagent (GE Healthcare Life Sciences), and luminescence signals were detected using the Fujifilm FPM-100A film processor (Fujifilm).

To determine the molecular mass of the recombinant proteins, 5 μg of recombinant SSC4D, SSC4D-d1d2, and SSC4D-d3d4 were run on SDS-PAGE, and proteins were detected by Coomassie blue staining; also, 0.5 μg of each recombinant protein were detected by western blotting.

### Cells and Cell Lines

Human peripheral blood mononuclear cells (PBMCs) were obtained from buffy coats of healthy adult volunteers at Banco de Sangue, Hospital São João, Porto, and were separated by Lymphoprep density gradient (STEMCELL Technologies). CD14^+^ monocytes were then isolated by positive magnetic cell sorting using CD14 microbeads (Miltenyi Biotec).

Differentiation of *ex vivo* monocytes into macrophages was achieved using 30 ng/ml of macrophage colony-stimulating factor (M-CSF) for 6 days in culture. Macrophages were then polarized toward an M1-like phenotype with 100 ng/ml lipopolysaccharide (LPS; *Escherichia coli* O111:B4; Sigma) and 25 ng/ml interferon (IFN)-γ (PeproTech), an M2a-like phenotype using 20 ng/ml interleukin (IL)-4 (PeproTech), or an M2c-like phenotype using 25 ng/ml IL-10 (PeproTech), all for 24 h. Polarization of undifferentiated monocytes was done similarly but for 72 h. Cell surface labeling using CD80 APC (2D10), CD206 PE (15.2), and CD163 BV421 (6H1/61) mAbs (all from BioLegend) confirmed the polarization of monocytes and macrophages into the correct subtype. Treatments with CD5L (1 μg/ml) or SSC4D (1 μg/ml) were assayed to check whether either of these stimuli would polarize cells toward any given subtype. Data were acquired in FACSCanto II (BD Biosciences). Post-acquisition analysis was performed using FlowJo software v10 (Tree Star).

Cell lines used in this study were Hep G2 (24), K562 (25), Caco-2 (26), E6.1 (27), JEG-3 (28), HEK 293T (29), TCCSUP (30), Raji (31), HL-60 (32), THP-1 (33), and HeLa (34). All lines were maintained at 37°C in a 5% CO_2_ humidified incubator in RPMI 1640 supplemented with 10% fetal calf serum (FCS), 1 mM sodium pyruvate, 2 mM L-glutamine, 50 U/ml penicillin G, and 50 μg/ml streptomycin, except HEK 293T, HeLa, Hep G2, and Caco-2 that were grown in Dulbecco’s modified Eagle’s medium (DMEM)/high-glucose medium containing 10% FCS, 1 mM sodium pyruvate, 4 mM L-glutamine, 50 U/ml penicillin, and 50 μg/ml streptomycin.

### Flow Cytometry and Cell Sorting

Blood from buffy coats was added to red blood cell (RBC) lysis buffer (BioLegend), and after washing, 1 × 10^6^ leukocytes were incubated with FcR blocking (Miltenyi Biotec) for 10 min at 4°C. Cells were stained with mAbs CD14 Pacific Blue (63D3), CD177 APC/Cy7 (MEM-166), CD19 PE/Cy7 (HIB19), CD4 Alexa Fluor 488 (OKT4), and CD8 APC (HIT8a) (all from BioLegend), fixed, and permeabilized with the eBioscience fixation/permeabilization kit (Thermo Fisher Scientific).

Intracellular staining was performed with rabbit anti-SSC4D polyclonal antibody, followed by anti-rabbit PE labeling (Life Technologies). Data were acquired in the FACSCanto II and post-acquisition analysis performed using FlowJo v10.

For cell sorting, blood from buffy coats was added to RBC lysis buffer, and 1 × 10^7^ leukocytes were stained with mAbs CD14 APC (63D3), CD177 APC/Cy7 (MEM-166), CD19 PE/Cy7 (HIB19), and CD3 PerCP/Cy5 (OKT3). The labeled cells were sorted with FACSAria (BD Biosciences).

### Immunostaining

SSC4D protein expression was detected in sections of human tissues kindly provided by the Unidade Local de Saúde de Matosinhos–Hospital Pedro Hispano. All ethical and legal issues were secured, along with the guarantee of confidentiality/no disclosure or violation of personal information or other data of the patients.

Four-micrometer sections of paraffin-embedded human blocks were deparaffinized and hydrated. Antigen retrieval was performed in 10 mM sodium citrate buffer for 30 min in a 96°C water bath.

Immunohistochemistry (IHC) was performed using UltraVision Quanto Detection System HRP DAB (Thermo Scientific). Endogenous peroxidase activity and nonspecific background staining were blocked using Hydrogen Peroxidase Block and Ultra V Block reagents, respectively. Tissues were immunostained with mouse anti-human SSC4D mAb 46-M or with a negative control normal mouse IgG sc-2025 (Santa Cruz Biotechnology) at 4°C overnight (ON), incubated with the primary antibody amplifier for 10 min followed by incubation with HRP Polymer Quanto and developed with 3, 3'-diaminobenzidine (DAB). The slides were counterstained with hematoxylin and visualized under light microscopy.

Colon, stomach, and liver sections were analyzed by immunofluorescence (IF). Non-specific staining was blocked with PBS containing 1% bovine serum albumin (BSA) for 1 h at room temperature (RT). Slides were then immunostained at 4°C ON with rabbit anti-SSC4D polyclonal followed by incubation with goat anti-rabbit Alexa Fluor 488-conjugated antibody (Life Technologies) for 1 h at RT. Nuclei were stained with "4′,6-diamidino-2-phenylindole (DAPI)" (Invitrogen), and cell preparations were mounted with Vectashield mounting media (Vector Laboratories). The slides were analyzed using confocal microcopy (Leica TCS SP5).

Fluorescence-activated cell sorting (FACS)-separated blood cells were adhered to poly-L-lysine (Sigma-Aldrich)-treated coverslips followed by blocking of non-specific staining with PBS containing 1% BSA for 1 h at RT. SSC4D was then detected with rabbit anti-SSC4D antibody ON at 4°C followed by incubation with goat anti-rabbit Alexa Fluor 488- conjugated antibody for 1 h at RT. Nuclei were stained with DAPI, and cell preparations were mounted with Vectashield. The slides were analyzed using confocal microcopy.

### Bacteria and Parasites

*E. coli* strains [BL21(DE3), IHE3034, RS218, and CFT073] were kindly provided by Claire Poyart (Institut Cochin, Paris), *Listeria monocytogenes* strain EGD-e and *Salmonella enterica* serovar typhimurium were provided by Didier Cabanes (i3S, Porto), and *Streptococcus agalactiae* [group B streptococcus (GBS)] strain BM110 was provided by Patrick Trieu-Cuot (Institut Pasteur, Paris). *Klebsiella pneumoniae*, *Enterococcus faecalis*, and *Pseudomonas aeruginosa* were also used in this study. Bacteria were grown to mid-logarithmic phase (OD_600_ of 0.45) in brain heart infusion medium at 37°C. *Mycobacterium avium* strain 2447 was prepared as described previously (35).

Parasites were prepared as previously described: *Neospora caninum* tachyzoites (Nc-1, ATCC 50843) (36), *Plasmodium berghei* ANKA strain blood merozoites (clone 676cl1) (37), *Trypanosoma brucei brucei* Lister 427 bloodstream forms (38), *Leishmania major* strain LV39, and *Leishmania tarentolae* strain Parrot-TarII promastigotes (39). A green fluorescent protein (GFP)-expressing *T. brucei brucei* Lister 427 line was engineered by cloning an enhanced *gfp* into a modified pHD1034 vector where the puromycin resistance cassette was replaced by the hygromycin resistance from the pHD1145 vector. Transfected parasites were selected with 5 µg/ml hygromycin (38).

### Scavenger Receptor Cysteine-Rich Protein–Microbial Cell Binding Assays

Binding of SRCR proteins to microbial cells was performed as described previously (17) using 2 μg of each protein interacting with 1 × 10^8^ live bacteria or 1 × 10^7^ live parasites in binding medium (TBS with 1% BSA, 5 mM Ca^2+^) for 1 h in an orbital shaker at 4°C. Microbe-bound proteins were detected using mouse anti-Tetra HIS mAb (Qiagen) followed by incubation of anti-mouse HRP-conjugated antibody (BioLegend) for 1 h at RT. Immunoblots were developed using ECL. Sample loading was evaluated with a rabbit anti-*Leishmania infantum* cysteine synthase (at 1:2,000 dilution) for *Leishmania* parasites and a rabbit anti-*T. brucei* aldolase (1:5,000) for *T. brucei*.

For the visualization of SSC4D binding to *T. brucei* bloodstream forms by IF, a GFP-expressing *T. brucei* Lister 427 line was incubated with 2 μg of HA-tagged SSC4D-FL in the binding medium. The cell pellet was transferred onto poly-L-lysine-treated coverslips and fixed with PFA 4% for 15 min at RT, and the presence of SSC4D was detected using anti-HA mAb 16B12 (BioLegend) followed by incubation with anti-mouse Alexa Fluor 568-conjugated antibody (Invitrogen). Nuclei were stained with DAPI, and the preparations were mounted with Vectashield. The slides were analyzed using confocal microcopy.

### Scavenger Receptor Cysteine-Rich Protein–Endotoxin Binding Assays

High-binding 96-well microtiter plates were coated ON with 10 μg/ml of purified LPS (*E. coli* O111:B4; Sigma) or 10 μg/ml lipoteichoic acid (LTA; Staphylococcus aureus; Sigma) in PBS at 4°C. The plates were blocked with PBS, 1% BSA, for 1 h at RT. Serial 2-fold dilutions of HIS-tagged SRCR proteins were added to the plates and incubated for 2 h at RT. Bound proteins were detected using mouse anti-HIS mAb for 1 h at RT, followed by goat anti-mouse HRP-conjugated antibody for 1 h at RT. Reactions were developed using SIGMAFAST o-Phenylenediamine dihydrochloride (OPD) for 30 min at RT and stopped with 1 M H_2_SO_4_. Absorbance was read at 490 nm using Synergy 2 (BioTek).

To calculate the calibration of the LPS- and LTA-binding assays, samples of serially diluted HIS-tagged SRCR proteins were directly coated on 96-well microtiter plates ON in PBS at 4°C. Plates were blocked with blocking solution for 1 h at RT, followed by detection of bound protein, as described above.

In between each step, plates were washed four times with PBS, 0.1% Tween-20.

### Scavenger Receptor Cysteine-Rich Protein–Eukaryotic Cell Binding Assays

To detect binding of SSC4D to putative ligands in eukaryotic plasma membranes, 2 × 10^5^ primary monocytes or Caco-2, Hep G2, Raji, K562, HL-60, THP-1, HeLa, or E6.1 cells were incubated with 3 μg of recombinant soluble extracellular CD6 (sCD6) or SSC4D-FL, or left untreated, in binding medium for 1 h at 4°C. Cells were then washed twice and incubated with fixable viability dye (Invitrogen) to exclude dead cells. The presence of SRCR proteins was detected with anti-HIS primary antibody followed by incubation with anti-mouse Alexa Fluor 647-conjugated antibody (Invitrogen) at 4°C. Data were acquired in FACSCanto II, and post-acquisition analysis was performed using FlowJo v10.

### Phagocytosis Assays

Monocytes were plated at a density of 2 × 10^5^ cells/well using imaging media (RPMI without phenol red, 10% FBS, 50 U/ml penicillin, and 50 μg/ml streptomycin) in 96-well plates (CellCarrier Ultra, PerkinElmer). After ON incubation, imaging media were removed without disturbing the monolayer and replaced with new media containing Hoechst for 45 min at 37°C. Then, 40 μg/ml of Invitrogen™ pHrodo™ Red *E. coli* BioParticles™ (Fisher Scientific) were added to the cells alone or with 5 μg/ml of recombinant SSC4D or CD5L. Immediately after, the 96-well plates were inserted in the IN Cell Analyzer (GE Healthcare Life Sciences), previously heated for 37°C, and nine images per well were collected 45 and 120 min after the addition of the BioParticles. Images were analyzed using FIJI software, and the percentage of cells containing *E. coli* BioParticles was determined.

### SSC4D Secretion Upon Infection of Caco-2 Cells

Caco-2 cells expressing an SSC4D-citrine-HA fusion protein were plated at a density of 3.5 × 10^5^ cells/well in 12-well plates. After cell attachment, cultures were infected for 1 h with live *E. coli* RS218 or *L. monocytogenes* EGD-e with a multiplicity of infection (MOI) of 1:50 or left uninfected. Cells were then washed with PBS and supplied with new media containing 20 μg/ml gentamicin. Supernatants were collected 2, 8, and 24 h after infection and resuspended in Laemmli’s sample buffer for SDS-PAGE and western blotting. SSC4D from supernatants was detected using mouse anti-HA mAb followed by anti-mouse HRP-conjugated antibody and ECL detection.

## Results

### Human SSC4D Protein Structure and Expression

**Figure 1:**
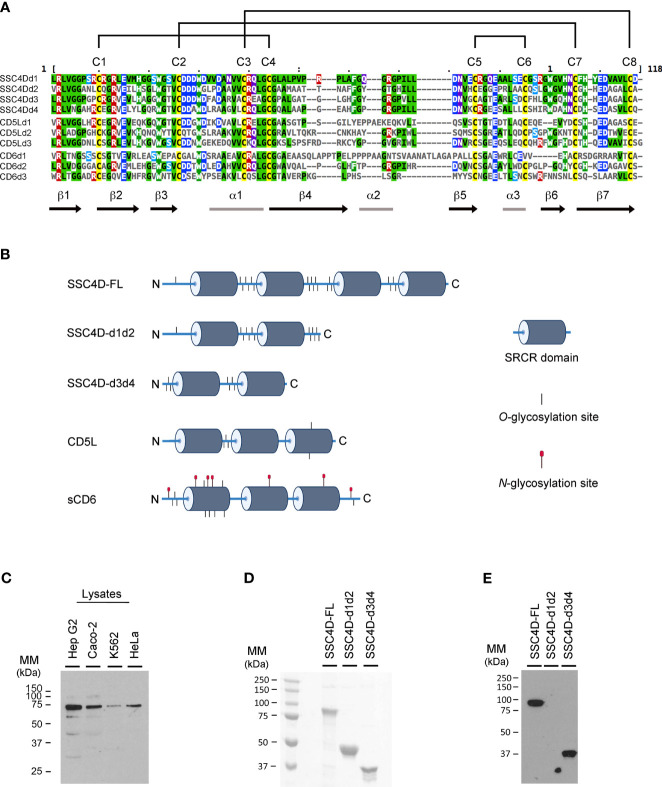
Amino acid sequence and structure of group B scavenger receptor cysteine-rich (SRCR) domains of SSC4D, CD5L, and CD6. **(A)** SRCR domains are typically ~100–110 amino acids in length compacted into a heart-shaped fold, where a six/seven-stranded β sheet cradles a core α1-helix. Each line represents one SRCR domain of the indicated protein. Amino acid sequences were aligned using Clustal Omega and MView (40). Amino acid side chain color codes for conserved residues: Green/black, aliphatic; Green/white, aromatic; Blue, anionic; Red, cationic; Cyan, polar; Magenta, amide; Yellow, sulfur-containing. Intrachain disulfide bonds established between conserved cysteine residues are shown on the top by connecting lines. **(B)** Schematic representation of the protein structures of SSC4D-FL, SSC4D-d1d2, SSC4D-d3d4, CD5L, and extracellular domain of CD6 (sCD6). SRCR domains are represented as dark cylinders. Putative *O*-linked glycosylation sites are represented as short vertical lines and *N*-linked glycosylation sites as lines topped with red circles. N and C termini of the proteins are indicated by “N” and “C,” respectively. Design was created using BioRender.com. **(C)** SSC4D protein expression detected by western blotting from cell lysates of Hep G2, Caco-2, K562, and HeLa cells. The molecular mass of intracellular SSC4D was calculated as 70.8 kDa. **(D)** Recombinant SSC4D, SSC4D-d1d2, and SSC4D-d3d4 were run on sodium dodecyl sulfate polyacrylamide gel electrophoresis (SDS-PAGE), and gels were stained with Coomassie blue. The size of recombinant extracellular full-length SSC4D was measured as 90.6 kDa, SSC4D-d1d2 as 45 kDa, and SSC4D-d3d4 as 36 kDa. **(E)** Recombinant SSC4D, SSC4D-d1d2, and SSC4D-d3d4 were run on SDS-PAGE, transferred to nitrocellulose membranes, and detected by immunoblotting. SSC4D and SSC4D-d3d4 were confirmed at the correct sizes, while SSC4D-d1d2 is not detected given that the polyclonal antibodies recognize sequences within domains 3 and 4.

SSC4D belongs to the group B of SRCR domain-containing proteins characterized by having an extraordinary sequence similarity between all individual domains and a nearly perfect conservation of key residues, namely, eight regularly spaced cysteine residues that establish intra-domain disulfide bonds in very defined combinations, also sequences that are 100% conserved in all known domains, especially in the β1 and β2 strands and in the boundaries between the α1 helix and the β4 strand (14) (**Figure 1A**). One other characteristic feature of this family of extracellular proteins consists of its extended level of glycosylation, as assessed by the high number of putative *O*-GalNAc glycosylation sites, characteristic of mucins. In particular, the four SRCR domains of SSC4D are interspaced with sequences rich in *O*-linked sugars, as predicted by NetOGlyc 4.0 (41) (**Figure 1B**). However, a certain separation can be established between the SRCR group B members that are secreted from those that are membrane bound, such as CD5, CD6, CD163, and CD163 antigen-like 1 (M160), in that in this latter set, *N*-linked glycosylation seems to be more prevalent, despite that the whole level of sequence similarity between the different proteins would not predict that sort of cleavage (14, 42). In fact, neither SSC4D nor CD5L, which are here investigated, contain any *N*-linked sugars as predicted by NetNGlyc 1.0 (43), contrasting with the extracellular domain of CD6 that contains seven such modifications.

We assessed the expression of SSC4D in lysates of several human cell lines and detected by western blotting the expression of the SSC4D protein in Hep G2, Caco-2, K562, and HeLa cells, and the molecular mass of intracellular SSC4D was calculated to be 70.8 kDa (**Figure 1C**). Few smaller bands of lower intensity could be observed in the blots, and these could hypothetically represent alternative splicing-dependent isoforms. Indeed, a common property of most SRCR members is the generation of alternative splicing-dependent isoforms, many of them resulting in the absence of individual or multiple SRCR domains, as described for DMTB1, CD6, CD163, and M160 (44–46). Padilla et al. (20) had in fact detected by northern blotting different transcripts that could account for alternative SSC4D isoforms, and one *SSC4D* mRNA isoform described in a transcriptome-wide study does miss the sequences encoding domains 3 and 4 (44). However, it is unlikely that the smaller bands in the gel correspond to this isoform because the detecting antibody was raised against sequences within domains 3 and 4 of the protein; rather, they either are unspecific blot bands or may represent degradation products.

Nevertheless, for the purpose of this study, we generated and expressed recombinant human full-length SSC4D and two recombinant hemi-SSC4D forms, each corresponding to one-half of the molecule and consisting of either the SRCR domains 1 and 2 (SSC4D-d1d2) or 3 and 4 (SSC4D-d3d4) (**Figure 1B**). The recombinant proteins were run on SDS and stained with Coomassie blue (**Figure 1D**) and detected by western blotting using an anti-SSC4D-d3d4 polyclonal antibody (**Figure 1E**). The molecular mass of the mature full-length extracellular protein was measured at 90.6 kDa, suggesting that the secreted protein undergoes posttranslational modifications, possibly *O*-linked glycosylation.

### SSC4D Expression in Human Epithelia and Leukocytes

Based on the reported tissue distribution of the mRNA coding for human SSC4D (20), we screened by PCR different human cell lines for the presence of *SSC4D* mRNA. We found that *SSC4D* is mostly expressed in cell lines with epithelial morphology like Hep G2 (hepatocellular carcinoma), Caco-2 (colorectal adenocarcinoma), JEG-3 (placental choriocarcinoma), HEK 293T (adenovirus 5 DNA-transfected embryonic adrenal precursor cells), and HeLa (cervical adenocarcinoma), but not in TCCSUP (urinary bladder carcinoma) (**Supplementary Figure S1**). Also, *SSC4D* mRNA was detected in hematopoietic-derived cells such as K562 (myelogenous leukemia) and very faintly in E6.1 (acute T-cell leukemia), but not in Raji (Burkitt’s lymphoma).

**Figure 2:**
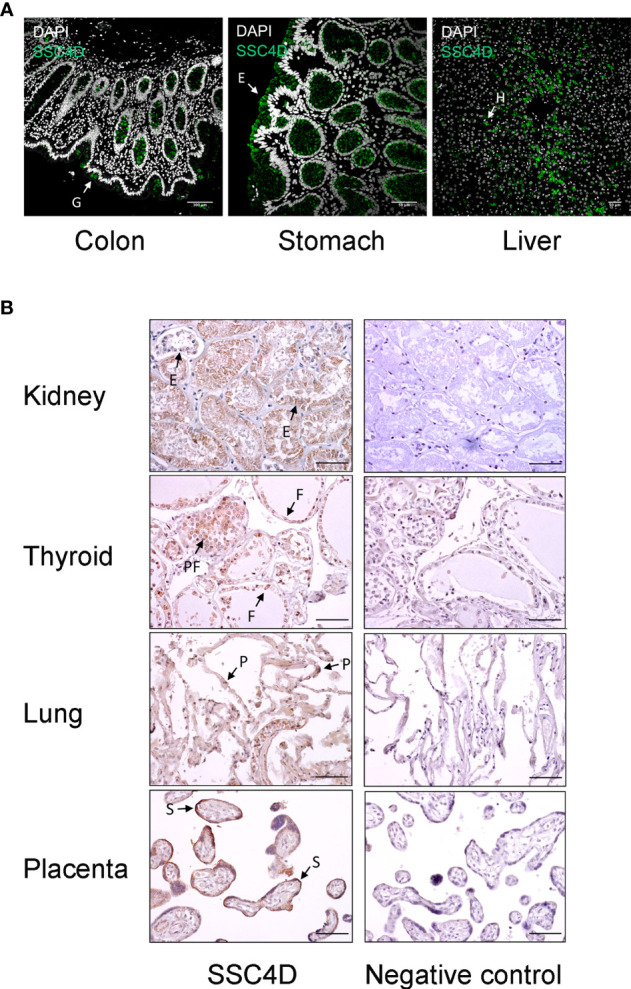
SSC4D distribution in human organs. **(A)** Detection of SSC4D by immunofluorescence (IF) in sections of the colon, stomach, and liver from normal human subjects. SSC4D labeling in mucous goblet cells (G) in the colon, simple columnar epithelium cells (E) in the stomach, and hepatocytes (H) in hepatic lobules is shown by arrows. Cell nuclei were stained with 4′,6-diamidino-2-phenylindole (DAPI; white). No unspecific staining was seen following incubation with secondary antibody alone, confirming specificity of the primary antibody. Scale bar, 50 μm. **(B)** Immunohistochemical analysis of SSC4D expression in sections of the kidney, thyroid, lung, and placenta. On the left column, SSC4D labeling was visualized by horseradish peroxidase (HRP) and substrate chromogen 3, 3'-diaminobenzidine (DAB). Positive staining of tubular epithelial cells (E) in the kidney, follicular (F) and parafollicular cells (PF) in the thyroid, pneumocytes (P) of the alveolar ducts, and of syncytiotrophoblasts (S) in the placenta is indicated by arrows. On the right column, images of sections labeled with unspecific mouse IgG mAb (negative control, sc-2025). Scale bar, 50 μm. Immunohistochemistry (IHC) and IF experiments were performed multiple times using samples from at least three different individuals.

We then assessed the expression of the protein in human organs. Relevant expression was observed in the gastrointestinal tract, with SSC4D being detected in intestinal crypts, namely, in mucous goblet cells in the colon, while in the stomach, staining was visualized in the simple columnar epithelium of the gastric mucosa and showing a broad distribution in gastric glands, compatible with SSC4D being expressed by surface mucous cells, mucous neck cells, and chief cells (**Figure 2A**). SSC4D was also expressed in the parenchyma of hepatic lobules in hepatocytes.

Regarding the genitourinary tract, strong SSC4D expression was detected in the epithelial cells of the tubules (**Figure 2B**). SSC4D was also found in follicular and parafollicular cells of the thyroid and in pneumocytes of the alveolar ducts. Interestingly, strong and specific expression of SSC4D was found in chorionic villi in placenta, mostly in the outer layer corresponding to the syncytiotrophoblasts.

**Figure 3:**
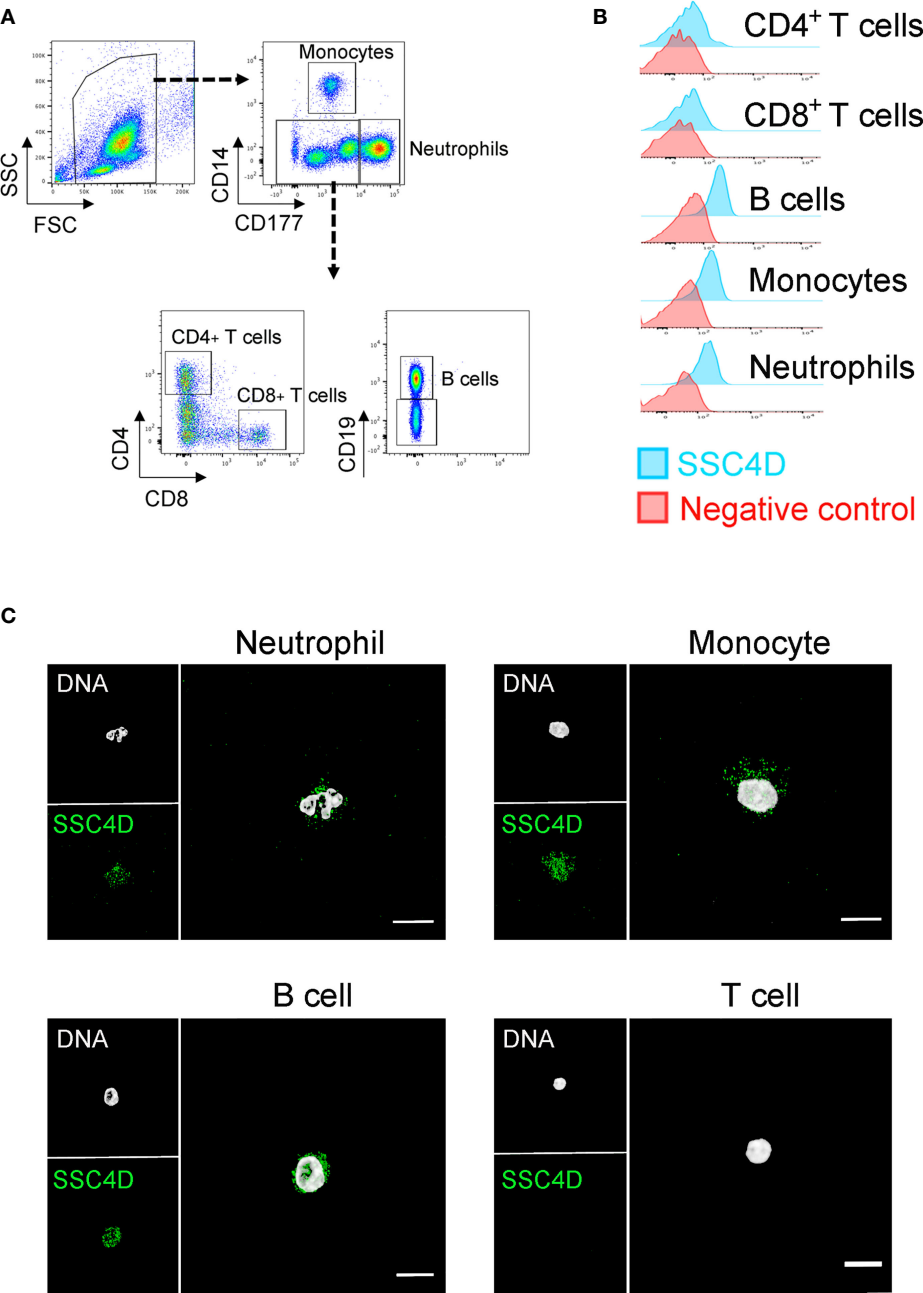
SSC4D expression in human leukocytes. **(A)** Flow cytometry gating strategy for the identification of monocytes, neutrophils, B cells, and CD4^+^ and CD8^+^ T cells from human blood. **(B)** Intracellular labeling of SSC4D in different human cell populations, visualized by flow cytometry. In the control samples, the anti-SSC4D antibody was omitted. Representative results shown are from one of four independent experiments. **(C)** Representative single-cell images of FACS-sorted leukocytes, immunostained for SSC4D (green) and visualized by immunofluorescence (IF). White indicates DAPI staining. Representative results shown are from one of three independent experiments using different donors.

We additionally assessed the expression of SSC4D in leukocyte subpopulations by flow cytometry and IF of FACS-sorted cells and detected the presence of intracellular SSC4D in monocytes, neutrophils, and B cells, but not in CD4^+^ or CD8^+^ T lymphocytes (**Figure 3**). Being a secreted protein, we questioned whether SSC4D could bind and exert any effect in target cells. For that purpose, we tested the binding of recombinant SSC4D to a panel of cell lines; however, none of the cells used were bound by SSC4D, whereas recombinant soluble extracellular CD6 (sCD6), used as a positive control, displayed the characteristic pattern of binding to cells that express its ligand, CD166 (47) (**Supplementary Figure S2**). This raises the possibility that SSC4D does not have a binding receptor at the surface of human cells or that a hypothetical receptor is not widespread.

### SSC4D Physically Binds to Gram-Positive and Gram-Negative Bacteria

The SSC4D-related proteins CD5L and SSC5D are able to identify a large spectrum of bacterial species and strains (16, 17); moreover, the ectodomain of CD6 was reported to bind and induce the aggregation of bacteria through the recognition of the bacterial endotoxins LTA and LPS (8). We investigated whether SSC4D could also detect different bacterial species and how the strength of interactions would compare with those of other SRCR family members.

**Figure 4:**
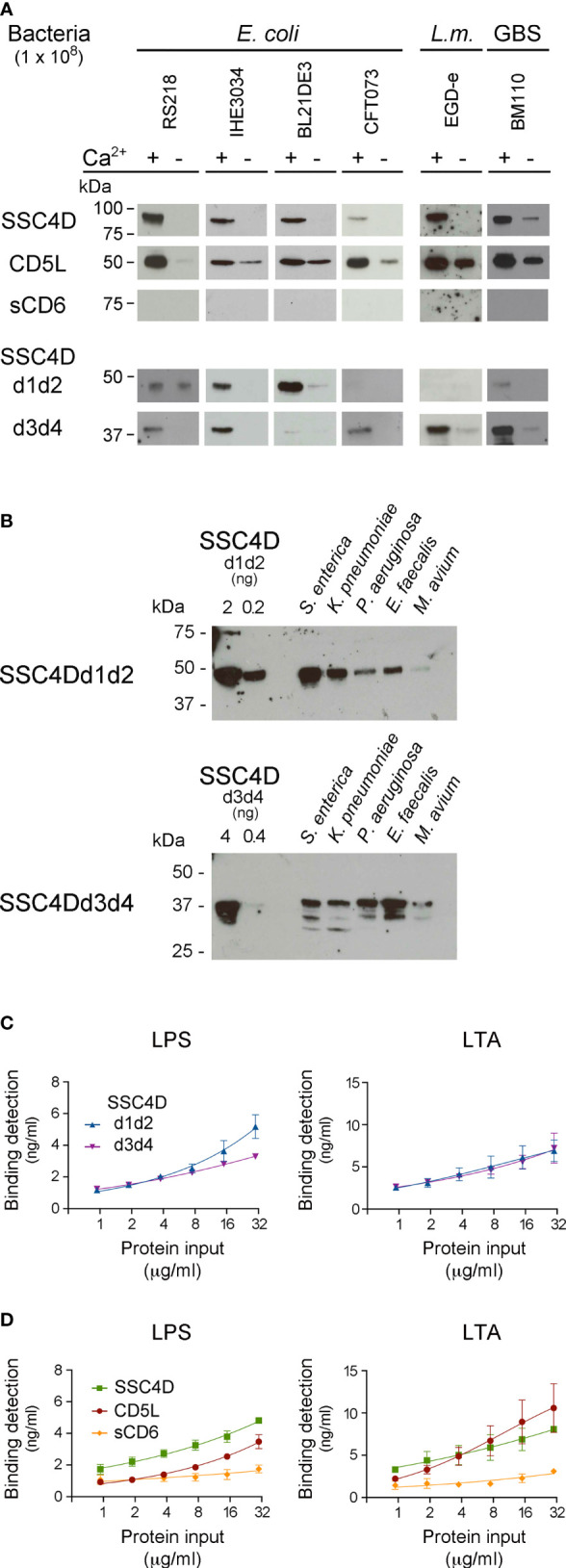
SSC4D physically binds to bacteria and bacterial endotoxins. **(A)** Two micrograms of each recombinant protein SSC4D, CD5L, sCD6, SSC4D-d1d2, and SSC4D-d3d4 were incubated with suspensions of 1 × 10^8^ CFU of live *Escherichia coli* strains BL21(DE3), RS218, IHE3034, or CFT073, *Listeria monocytogenes* strain EGD-e, or GBS strain BM110 in the presence or absence of Ca^2+^. Cell-bound proteins were detected by immunoblotting using anti-HIS mAb. Blots are representative of at least three independent experiments. **(B)** Recombinant SSC4D-d1d2 and SSC4D-d3d4 (2 μg each sample) were incubated with suspensions of 1 × 10^8^ CFU of live *Salmonella enterica*, *Klebsiella pneumoniae*, *Enterococcus faecalis*, *Pseudomonas aeruginosa*, or *Mycobacterium avium* in the presence of Ca^2+^. Bacteria-bound proteins were detected by immunoblotting using an anti-HIS mAb. The sensitivity of detection of this mAb for each of the recombinant hemi-forms of SSC4D can be evaluated by the detection, shown on the left side of the membranes, of 2 and 0.2 ng of purified SSC4D-d1d2 (upper blot) or 4 and 0.4 ng of purified SSC4D-d3d4 (lower blot). **(C)** Binding of SSC4D-d1d2 and SSC4D-d3d4 to plate-bound lipopolysaccharide (LPS) and lipoteichoic acid (LTA). Proteins were added at the indicated concentrations, and signals were detected by anti-HIS mAb followed by horseradish peroxidase (HRP)-conjugated antibody and o-Phenylenediamine dihydrochloride (OPD) substrate. Absorbance was read at 490 nm. Binding values shown were interpolated from standard curves of detection of plate-bound SSC4D-d1d2 and SSC4D-d3d4, shown in **Supplementary Figure S3**. Graphs show the mean ± SD of two independent experiments performed in duplicate. **(D)** Binding of SSC4D, CD5L, and sCD6 to plate-bound purified LPS and LTA. Detection and measurement of binding were as in panel **(C)**.

Recombinant SSC4D, CD5L, and sCD6 were incubated with samples of live *E. coli* strains BL21(DE3), IHE3034, and RS218, with *L. monocytogenes* EGD-e, and with GBS BM110, followed by centrifugation and immunoblotting of the pelleted bacteria. As anticipated, we observed a strong interaction between CD5L and all tested bacteria, particularly in the presence of calcium given that many SRCR protein-mediated interactions are Ca^2+^-dependent (**Figure 4A**). By contrast, the interactions between sCD6 and the different bacteria were not visually detectable. Importantly, SSC4D clearly bound all bacteria tested, demonstrating its ability to physically interact with conserved structures present at the surface of these microorganisms.

Consequently, we included in our bacteria binding assays the two recombinant hemi-SSC4D forms, SRCR-d1d2 and SRCR-d3d4. Performing the assays using the same bacterial samples, we observed in several cases that the two halves of the molecule had differential binding profiles, such that SSC4D-d3d4 bound well to *Listeria* and GBS, whereas binding of SSC4D-d1d2 to these bacteria was much less evident (**Figure 4A**, lower panels). Conversely, although not as clear as the above, it seemed that the *E. coli* strains, with the exception of *E. coli* CFT073, were better recognized by SSC4D-d1d2.

We hypothesized that each half of SSC4D might bind preferentially to different groups of bacteria and therefore increased the sampling of our assays by adding supplementary bacterial species. In each assay, recombinant SSC4D-d1d2 or SSC4D-d3d4 was incubated with live Gram-negative *S. enterica*, *K. pneumoniae*, and *P. aeruginosa* and with Gram-positive *E. faecalis* or *M. avium* (**Figure 4B**). Although there was not an absolute compartmentalization in the recognition profiles, in general, it appears that SSC4D-d1d2 displays a preference for binding Gram-negative bacteria. Although the converse correlation cannot be fully established for SSC4D-d3d4, as this half of the molecule is more homogeneous in the identification of both bacterial groups, it appears that SSC4D-d3d4 binds better to Gram-positive bacteria than does SSC4D-d1d2 (**Figure 4B**).

We thus evaluated whether each typical endotoxin of Gram-negative and Gram-positive bacteria would be a preferential target of one-half of the SSC4D molecule over the other using an ELISA to measure the affinity of each protein to LPS and LTA. We first assessed the sensitivity of the detecting antibody to plate-bound purified SRCR proteins (**Supplementary Figure S3A**), following which we measured the binding of serially diluted HIS-tagged SRCR proteins to microtiter plates coated with 10 μg/ml of purified LTA or LPS (**Supplementary Figure S3B**). The conversion of the obtained values to binding detection units showed that both SSC4D hemi-forms bound to LPS and LTA in a dose-dependent manner, but whereas in fact LPS was superiorly targeted by SSC4D-d1d2 than by SSC4D-d3d4 at higher protein concentrations, the plots for binding to LTA were indistinguishable between the two subunits (**Figure 4C**).

Comparing the binding forces to LPS and LTA between CD5L, SSC4D, and sCD6, again binding of the proteins to the endotoxins is differentiated: SSC4D binds to LPS with higher avidity than CD5L, whereas binding to LTA is not significantly different between these two proteins (**Figure 4D**). In accordance with the previous experiments and our earlier work (17), binding of sCD6 to either live or fixed bacteria, or to bacterial endotoxins, although detectable, is inferior when compared with the microbe-binding capacity of the natural circulating SRCR proteins.

### SSC4D Promotes Phagocytosis but Does Not Induce Macrophage Polarization

**Figure 5:**
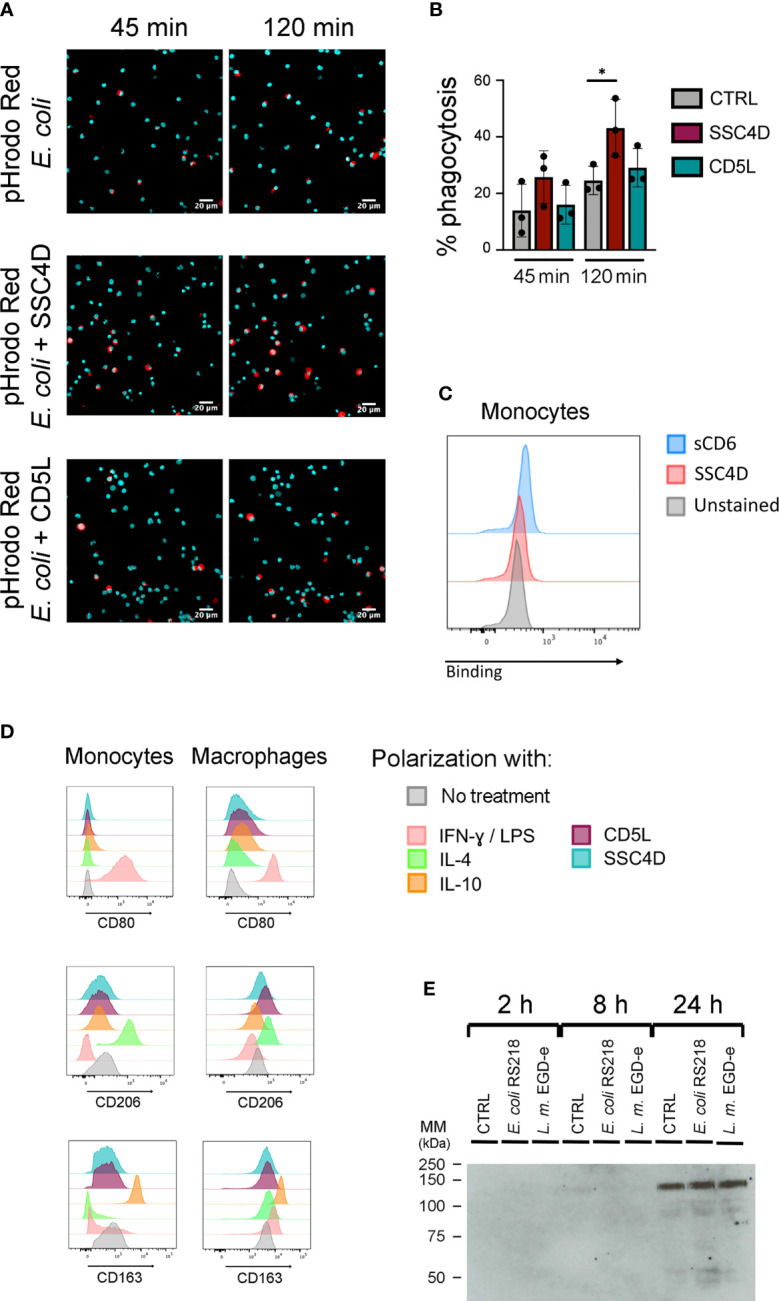
SSC4D promotes phagocytosis without binding to a ligand on human monocytes and does not induce macrophage polarization. **(A)** *Escherichia coli* pHrodo BioParticles (40 μg/ml) were added to isolated human monocytes, together with 5 μg/ml of SSC4D (middle panels) or CD5L (bottom panels), or no protein (top panels). Images were acquired for each well at 45 and 120 min after the addition of *E. coli* BioParticles using an IN Cell Analyzer, followed by analysis using FIJI software. Blue indicates DAPI staining, and red indicates phagocytosed *E. coli* BioParticles. **(B)** The percentage of monocytes containing *E. coli* BioParticles was quantified. Graph shows the mean ± SD of three independent experiments performed in duplicate. Statistical analysis was performed using Student’s t test. *p < 0.05. **(C)** *Ex vivo* monocytes were incubated with 3 μg of SSC4D or sCD6 or left unstained. Cell-bound proteins were detected with anti-HIS antibody followed with Alexa Fluor 647-conjugated anti-mouse antibody and analyzed by flow cytometry. Gray histograms represent control cells, not stained with scavenger receptor cysteine-rich (SRCR) protein but incubated with secondary antibody, red histograms represent labeling with SSC4D, and blue histograms represent labeling with sCD6. **(D)** Flow cytometry analysis of *ex vivo* monocytes (left column) and macrophage colony-stimulating factor (M-CSF)-differentiated macrophages (right column). Monocytes received for 72 h the appropriate stimuli to polarize toward M1 [interferon (IFN)-γ/lipopolysaccharide (LPS)], M2a [interleukin (IL)-4], or M2C (IL-10) subtypes. Macrophages received the same treatment, but for 24 h. CD80, CD206, and CD163 labeling confirms the polarization of monocytes and macrophages into the correct subtype. Stimulations with SSC4D or CD5L had no effect on cell polarization except for a slight effect of CD5L in polarizing macrophages into an M2a-like phenotype. Representative histograms are from one of three independent experiments. **(E)** SSC4D secretion upon culture infection with live bacteria. Caco-2 cells were engineered to express SSC4D fused to citrine and were cultured for 3 days at 3 × 10^5^ cells/well in a 12-well plate. Live *E*. *coli* RS218 or *L. monocytogenes* EGD-e were added at 1:50 multiplicity of infection (MOI). Supernatants were collected at the indicated time points, and the presence of HA-tagged SSC4D-citrine was detected by western blotting. The blot shown is representative of two independent experiments.

Because SSC4D is produced by phagocytes and binds to bacteria, we questioned whether this circulating molecule could have a direct impact on pathogen clearance. To measure protein-mediated phagocytosis, monocytes were incubated with pHrodo™ Red *E. coli* BioParticles™ in the presence of CD5L or SSC4D or in the absence of the recombinant proteins. These BioParticles become fluorescent in acidic pH, only identifying those bacteria that are inside phagosomes (48). Monocyte phagocytosis of the *E. coli* particles increased over time but was not different whether CD5L was present or not (**Figures 5A, B**). By contrast, the presence of SSC4D induced a significant increase in the phagocytic capacity. To test whether SSC4D could mediate the internalization of the bacteria through an interaction to a putative cellular receptor in the phagocyte, we checked for a direct interaction between recombinant SSC4D and monocytes. However, as can be seen in **Figure 5C**, no such interaction is obvious, whereas sCD6, used as a binding control, is able to interact slightly with monocytes, given that these cells express low levels of CD166. An alternative explanation is that the increase in phagocytosis could be due to increased activation of monocytes induced by SSC4D. Although conceivable, this possibility is unlikely, given that SSC4D was added to the cells at the same time as the *E. coli* particles and the duration of the experiment was perhaps too short to allow for a vigorous monocyte activation-mediated phagocytosis.

Also displaying opposite effects from CD5L, SSC4D did not induce the polarization of macrophages toward an M2 phenotype (**Figure 5D**). Differentiation of *ex vivo* monocytes with CD5L for 3 days had an equivalent result as utilizing IL-4 in the development of an M2a-like phenotype, as previously reported (49). However, in no other differentiation and polarization protocol did SSC4D, nor CD5L, induce monocyte/macrophage polarization, including no effect on an M1-type phenotype.

SSC4D is normally detected in cell culture media at very low levels, so we questioned whether SSC4D secretion could escalate due to any type of immune response and what would be the external cues that could stimulate this secretion. Caco-2 cells that were engineered to produce a chimeric protein consisting of SSC4D fused to mCitrine and an HA-tag sequence (**Supplementary Figure S4A**) were incubated with live *E. coli* RS218 or *L. monocytogenes* EGD-e at a 1:50 MOI or left uninfected. Culture supernatants were collected at different time points, and the presence of SSC4D was assessed by western blotting. As seen in **Figure 5E**, secreted SSC4D was detected at 24 h post infection, but there were no differences between infected (with *E. coli* or *L. monocytogenes*) and non-infected cultures. It is possible that in this specific case, the detection of SSC4D in the media could result from cell death instead of induced, or passive, secretion; nonetheless, in all other tested conditions using different immune-inflammatory mediators or bacterial endotoxins to stimulate SSC4D secretion, there was no single specific stimulus that would further augment the rate of secretion (**Supplementary Figures S4B, C**). Instead, SSC4D was being continuously released into the medium at moderate levels, independently of any tested external cues.

### SSC4D Physically Binds to Protozoan Parasites

**Figure 6:**
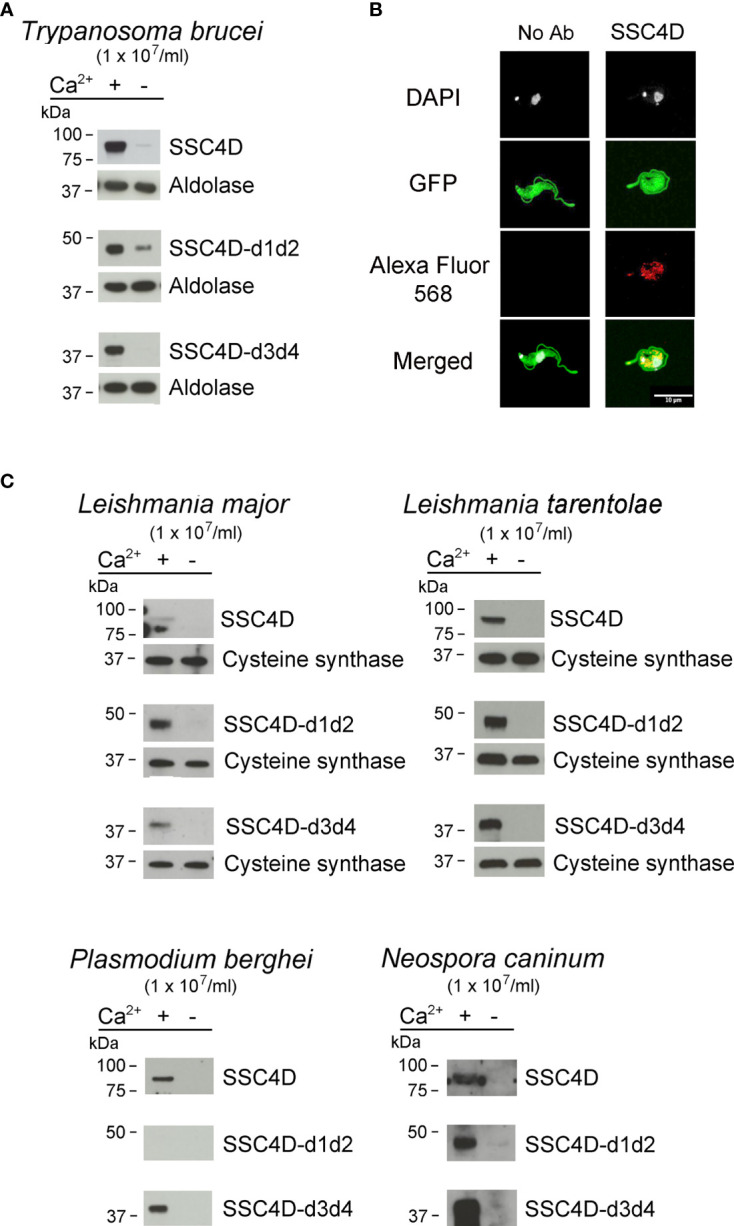
SSC4D binds to protozoan parasites. **(A)** Two micrograms of recombinant SSC4D, or of the hemi-forms SSC4D-d1d2 and SSC4Dd3d4, were incubated with suspensions of 1 × 10^7^ live *Trypanosoma brucei brucei* bloodstream forms in the presence or absence of Ca^2+^. Parasite-bound proteins were detected by immunoblotting using anti-HIS mAb. Membranes were reprobed with an anti-aldolase immune serum for loading control. Results shown are of one of three independent experiments. **(B)** Representative images of SSC4D interacting with green fluorescent protein (GFP)-expressing *T. brucei*. In both panels, GFP^+^ parasites (green) were allowed to interact with SSC4D (red), being the primary antibody omitted in the left panel, as control. DAPI (white) indicates DNA staining. The results shown are representative of four independent experiments. **(C)** Two micrograms of recombinant SSC4D, SSC4D-d1d2, and SSC4D-d3d4 were incubated with suspensions of 1 × 10^7^ live *Leishmania major* and *Leishmania tarentolae* promastigotes, *Plasmodium berghei* merozoites, and *Neospora caninum* tachyzoites. Interactions were detected as in panel **(A)**, and membranes were reprobed with an anti-*L. infantum* cysteine synthase immune serum for loading control of *L. major* and *L. tarentolae*.

PRRs are able to recognize not only bacterial but also fungal, viral, or protozoan conserved structural components. In order to test whether the binding properties of SSC4D could be expanded to protozoan targets, protein binding assays were performed with live parasites. We first assessed the binding of SSC4D to bloodstream forms of *T. brucei*, the parasite that causes African trypanosomiasis. Recombinant SSC4D was incubated with 1 × 10^7^ parasites, followed by centrifugation and immunoblotting of the cell pellet. As illustrated in **Figure 6A**, full-length SSC4D and each SSC4D half were capable of physically interacting with the parasite in a Ca^2+^-dependent manner. To image this interaction by confocal microscopy, a GFP-expressing *T. brucei* Lister 427 strain was incubated with HA-tagged SSC4D, and binding of the protein to *T. brucei* cells was detected using anti-HA mAbs (visualized in red, **Figure 6B**).

We extended our protein–parasite binding assays to *Leishmania major* and *Leishmania tarentolae* promastigotes, *Plasmodium berghei* merozoites, and *Neospora caninum* tachyzoites. SSC4D and its half-forms bound to all tested parasites, with the exception of SSC4D-d1d2 that did not bind to *P. berghei* merozoites, the stage that infects red blood cells. Noteworthy, in the absence of Ca^2+^, all SSC4D–parasite interactions were abolished or markedly reduced.

## Discussion

In this study, we show for the first time the capacity of SSC4D to physically bind to bacteria and protozoan parasites. SSC4D has been one of the most neglected SRCR proteins, and no functional data were available, but by simple analogy with other family members, we anticipated that this protein could reveal some PRR functions. The identity of bacterial targets of SSC4D does not significantly differ from those of CD5L or SSC5D; however, these proteins do not display identical binding patterns between themselves or even among their own single domains. We have previously shown relevant differences of binding avidities between SSC5D and CD5L toward different types of bacteria (17). We here advance on this conclusion by showing that different parts of SSC4D have preferential binding toward different groups of bacteria.

Contrasting with the strong binding of CD5L, SSC4D, and SSC5D to a variety of bacterial species and strains, the extracellular domain of CD6 displays a significantly lower binding potency. CD6 is a plasma membrane glycoprotein that modulates T-cell activation (23), and it was somewhat unexpected that such a receptor involved in antigen-dependent signal transduction would be directly involved in the recognition of unprocessed pathogenic determinants (8). Although there was some controversy as to which extent CD6 binding to bacteria would reflect a physiological characteristic of the molecule (50, 51), it seems undisputable that CD6 does protect from bacterial infection-induced septic shock in mouse models possibly *via* its function as a circulating extracellular form (sCD6), shed from the surface of lymphocytes in pathological conditions (52). Nonetheless, the fact that the levels of bacterial binding of SSC4D, like CD5L and SSC5D, are so much more evident than those of sCD6 clearly suggests that a main function of SSC4D is indeed of pathogen pattern recognition.

SSC4D is expressed by many epithelial cells of several organs and by phagocytic leukocytes, but unlike what has been described for other circulating SRCR proteins, we could not identify any stimulus, cue, or microbial challenge that increased the rate of secretion of the protein. The estimated plasma concentration of SSC4D is in effect very low (1 ng/ml) when compared with those of the other circulating SRCR proteins SSC5D (88 ng/ml), CD5L (4.3 μg/ml), and MAC2BP (7.1 μg/ml) (21, 22), and the abundance of these proteins further increases upon certain inflammatory and infectious challenges or in oncological environments (11, 53, 54). Also, the membrane-bound receptors CD5, CD6, and CD166, expressed by different leukocytes, undergo cleavage of their ecto-domains in particular pathological conditions, resulting in their consequent release into circulation where they display specific immune-related functions (55, 56). And yet, we have not found any similar agonist-dependent behavior for SSC4D, raising the possibility that SSC4D is being continuously secreted at low constant rates either in steady-state or upon external challenges.

Therefore, and despite sharing common functions with other SRCR proteins, namely, as a PRR, SSC4D may be endowed with some distinctive properties. SSC4D behaves differently from CD5L in at least a few aspects, as in contrast with CD5L (16, 49), SSC4D is not involved in the polarization of macrophages upon different inflammatory stimuli. On the other hand, SSC4D can potentiate the phagocytosis of bacteria by macrophages, contrary to human CD5L. Although our results on CD5L concur with those previously reported by Sanjurjo et al. (49), there is some controversy regarding the role of CD5L in phagocytosis, which may depend on the experimental setup, the type of particle/microorganism to be internalized, and the molecular and cellular species analyzed. Mouse (m)CD5L was shown to increase the phagocytosis of latex beads by mouse macrophages (57); both mCD5L and human (h) CD5L increase the clearance of debris by mouse macrophages (58); hCD5L increases clearance of apoptotic cells by human monocytes (49); and mCD5L increases phagocytosis by mouse macrophages and neutrophils of *S. aureus* (59). However, the presence of hCD5L did not change the phagocytosis of microspheres or *E. coli* or *S. aureus* particles by human peripheral blood cells (49).

We here show that phagocytosis of *E. coli* particles by human monocytes is in fact not influenced by CD5L but is increased in the presence of SSC4D. Given that both *E. coli* particles and SRCR proteins were added to the cells at the same time, it is unlikely that the increase in phagocytosis is due to activation of monocytes induced by SSC4D. It is possible, instead, that the protein intermediates the interaction between monocytes and bacteria. We have screened monocytes with recombinant monovalent SSC4D for the existence of specific receptors and could not detect any interaction by flow cytometry possible due to the low sensitivity of the method. Although with no evidence that SSC4D promotes large-scale bacterial aggregation, an alternative hypothesis is that the coating of bacteria with SSC4D may induce a more efficient recognition of multivalent SSC4D opsonizing the bacteria either by low-affinity SSC4D receptors or eventually by other sensors of microbial structures.

It is known that host defense against protozoan parasites involves different classes of PRR, such as TLRs, C-type lectin receptors, and NOD-like receptors (60–62). Nevertheless, the knowledge on this field lags considerably behind those that focus on the identification of bacterial, viral, and fungal PAMPs. Also, many other components of the innate immune system participate in antiparasitic defenses, including CD36, a scavenger receptor class B that displays multiple functions and a broad range of ligands, including a cytoadherence ligand on *Plasmodium falciparum*-infected erythrocytes (63). However, CD36 belongs to a different family of scavenger receptors characterized by having two transmembrane domains flanking a CD36-type multifunctional domain. SRCR proteins like MARCO and MSR1 have been shown to have a role in defense against protozoan parasites such that, for example, inhibition of MSR1 function reduces *P. berghei* infection and the expression of MARCO in macrophages of CBA/J mice is increased upon *L. major* infection (64, 65). Still, to the best of our knowledge, ours is the first study that describes a physical interaction of an SRCR protein and protozoan targets. Together with its capacity to bind bacteria and to promote macrophage phagocytosis, SSC4D can thus be considered a *bona fide* broad-range PRR, and importantly, this may help to strengthen the concept, so many times overlooked, that the SRCR cluster is a legitimate member of the wider collective family of pathogen PRRs.

## Data Availability Statement

The original contributions presented in the study are included in the article/**Supplementary Material**. Further inquiries can be directed to the corresponding author.

## Author Contributions

MC designed and performed the experiments and wrote the paper. RS performed IHC and flow cytometry. SA performed IHC and produced Caco-2-SSC4D cells. MS developed GFP+ parasites. BP-C performed protein–parasite assays. LO designed protein expression vectors, produced proteins, and handled bacterial experiments. JT designed all experiments handling parasites. AC planned and designed the study and wrote the paper. All authors contributed to the article and approved the submitted version.

## Funding

This work was funded by National Funds through FCT–Fundação para a Ciência e a Tecnologia, I.P., under the projects SRecognite Infect-ERA/0003/2015 and UIDB/04293/2020. Individual funding to JT was provided by FCT through CEECIND/02362/2017. MC, RS, and MS were recipients of studentships from FCT, respectively, SFRH/BD/116791/2016, SFRH/BD/110691/2015, and SFRH/BD/133485/2017.

## Conflict of Interest

The authors declare that the research was conducted in the absence of any commercial or financial relationships that could be construed as a potential conflict of interest.

## Publisher’s Note

All claims expressed in this article are solely those of the authors and do not necessarily represent those of their affiliated organizations, or those of the publisher, the editors and the reviewers. Any product that may be evaluated in this article, or claim that may be made by its manufacturer, is not guaranteed or endorsed by the publisher.
