# Supplementary material for: Physical Interactions With Bacteria and Protozoan Parasites Establish the Scavenger Receptor SSC4D as a Broad-Spectrum Pattern Recognition Receptor
Source: Front Immunol. 2021 Dec 24;12:760770. doi: 10.3389/fimmu.2021.760770 (PMC8739261; doi:10.3389/fimmu.2021.760770)
Supplement: Supplementary file 1 [file DataSheet_1.pdf]

SUPPLEMENTARY MATERIAL

METHODS

mRNA analysis of SSC4D in human cell lines, and expression of SSC4D-citrine fusion protein in Caco-2 cells

Total RNA of different cell lines was isolated using the TripleXtractor directRNA kit (Grisp). Using 5 μg of RNA per sample, cDNA was synthesized using Superscript III reverse transcriptase (Invitrogen). The cDNA obtained was used to analyze the SSC4D expression by PCR with GoTaq DNA Polymerase (Promega). Primer sequences were the following: forward, 5'-GGCGTCCACAATTGCTTTCA-3'; and reverse, 5'-ACGGATCTGTCTGCCAAG-3'.

Measurement of SSC4D accumulation in Caco-2 cells

SSC4D-expressing Caco-2 cells were plated at a density of 2 x 10^4 cells/well in a 96-well plate (CellCarrier Ultra). Cell seeding was optimized to achieve a confluency of 70% allowing optimal cell segmentation. Cells were maintained in imaging media and incubated for 3 days for optimal attachment. Culture media was then removed without disturbing the monolayer and replaced by fresh media containing Hoechst, for 45 min at 37 ºC.

For the 0 h time-point, cells were washed with sterile PBS and new medium was added, followed by image acquisition using the IN Cell Analyzer. A 20 x objective was used and 9 fields of view were collected in each well. Then, the plate was spun and different stimuli, IL-1β, IL-4, IL-6, IL-17, TNF-α, IFN-γ, LPS, and LTA, at different concentrations were added to the cells together with 10 μg/ml of Brefeldin A, an inhibitor of protein transport from the ER to the Golgi complex, and thus of protein secretion.

Image acquisition was done at 6 h post-stimuli, similar to the 0 h time-point. To quantify SSC4D intracellular accumulation in the Caco-2-SSC4D cells, first the nuclei of these cells were identified from the Hoechst channel, using a machine-learning-based (bio)image analysis tool – ilastik (66). The resulting pixel probability maps were used for further image analysis and quantification of the mCitrine intensity values per cell using another cell image analysis software – CellProfiler™ (67). Briefly, the image analysis workflow consisted in (i) correction for uneven

illumination/lighting/shading on the mCitrine channel, (ii) segmentation of the nuclei from the probability maps, (iii) expansion of the nuclei by 10 pixels to create a bigger mask that covers the majority of the cell cytoplasm, (iv) then the nucleus mask has been subtracted from the previous expanded mask and then (v) the mean pixel intensity per cell on the mCitrine channel has been quantified.

The mCitrine intensity for each cell was obtained and the average of for each field of view was then calculated. The intensity value of each field of view was normalized to the intensity value of the negative control (WT Caco-2 cells) followed by a normalization to the corresponding baseline condition (0 h).

Measurement of SSC4D secretion upon cytokine stimuli

Caco-2-SSC4D cells were plated at a density of 2 X 10^5 cells/well in a 12-well plate and incubated for 3 days for an optimum cell attachment. The culture medium was removed and new medium containing cytokines IL-1β, IL-4, IL-6, IL-17, TNF-α or IFN-γ in different concentrations (1, 10, and 100 ng/ml), or endotoxins LPS and LTA (at 10, 100, and 1000 ng/ml) was added to the cells. After the indicated times of incubation, supernatants were collected and then resuspended in Laemmli's sample buffer for SDS-PAGE and western blotting. The presence of SSC4D in the culture supernatants was detected using mouse anti-HA antibody followed by anti-mouse HRP conjugated antibody, and ECL detection.

REFERENCES

66. Berg, S., Kutra, D., Kroeger, T., Straehle, C. N., Kausler, B. X., Haubold, C., Schiegg, M., Ales, J., Beier, T., Rudy, M., Eren, K., Cervantes, J. I., Xu, B., Beuttenmueller, F., Wolny, A., Zhang, C., Koethe, U., Hamprecht, F. A., and Kreshuk, A. (2019) ilastik: interactive machine learning for (bio)image analysis. *Nat Methods* **16**, 1226-1232

67. McQuin, C., Goodman, A., Chernyshev, V., Kamentsky, L., Cimini, B. A., Karhohs, K. W., Doan, M., Ding, L., Rafelski, S. M., Thirstrup, D., Wiegraebe, W., Singh, S., Becker, T., Caicedo, J. C., and Carpenter, A. E. (2018) CellProfiler 3.0: Next-generation image processing for biology. *PLoS Biol* **16**, e2005970

Supplementary Figure 1

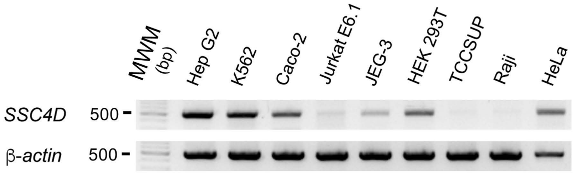

Supplemental Figure 1. *SSC4D* expression in human cell lines. Representative agarose gel images showing *SSC4D* mRNA expression in different human cell lines, measured by RT-PCR. *β-actin* mRNA is shown as loading control. MWM, molecular weight markers.

Supplementary Figure 2

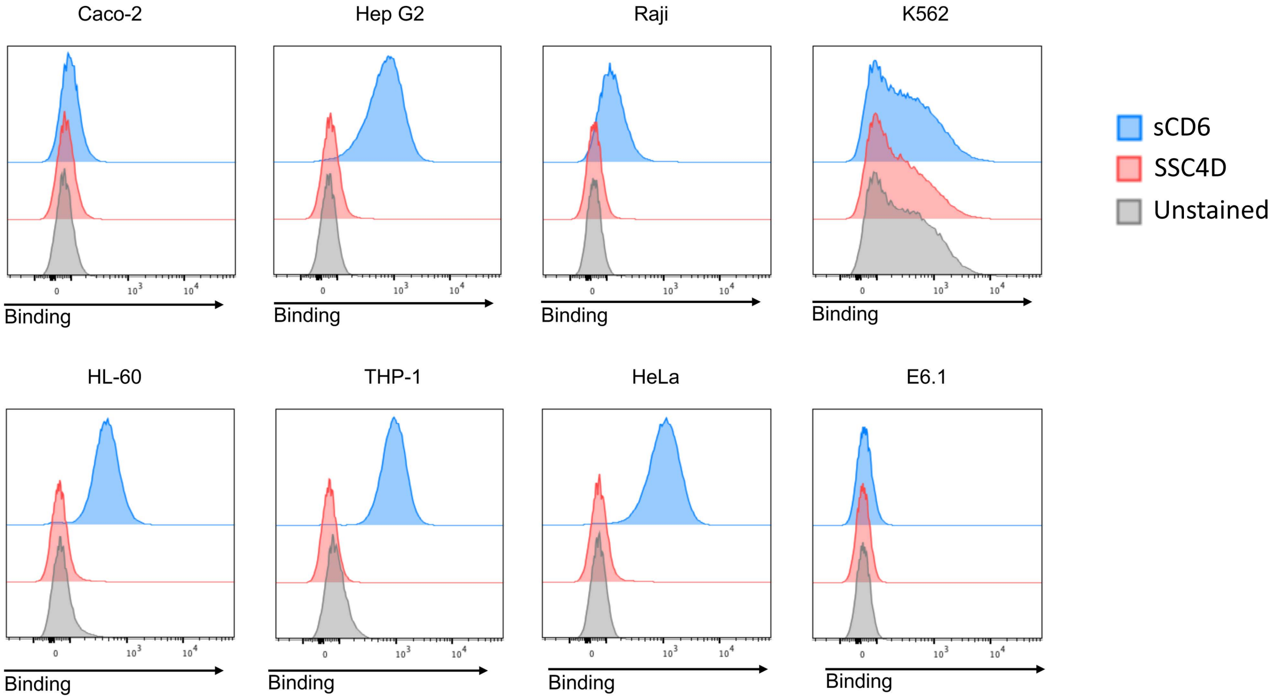

Supplemental Figure 2. No evidence for a cellular ligand for SSC4D. Caco-2, Hep G2, Raji, K562, HL-60, THP-1, HeLa and E6.1 cell suspensions were incubated with 3 μg of SSC4D or sCD6 or left untreated. Cell-bound proteins were detected with anti-HIS antibody followed with 647-conjugated anti-mouse antibody and analyzed by flow cytometry. Gray histograms represent control cells, not stained with SRCR protein but incubated with secondary antibody, red histograms represent cells labeled with SSC4D and blue histograms cells labeled with sCD6.

Supplementary Figure 3

A

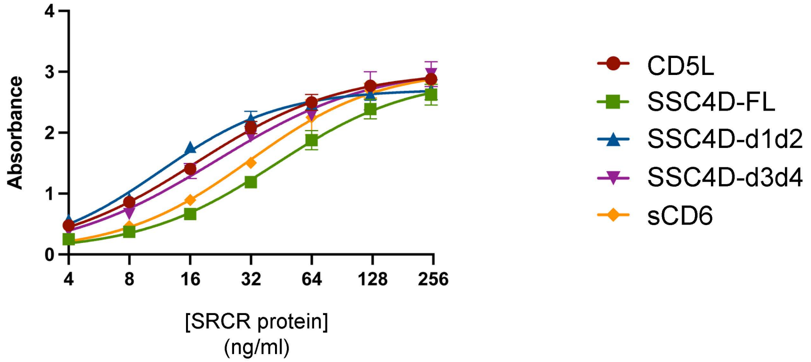

B

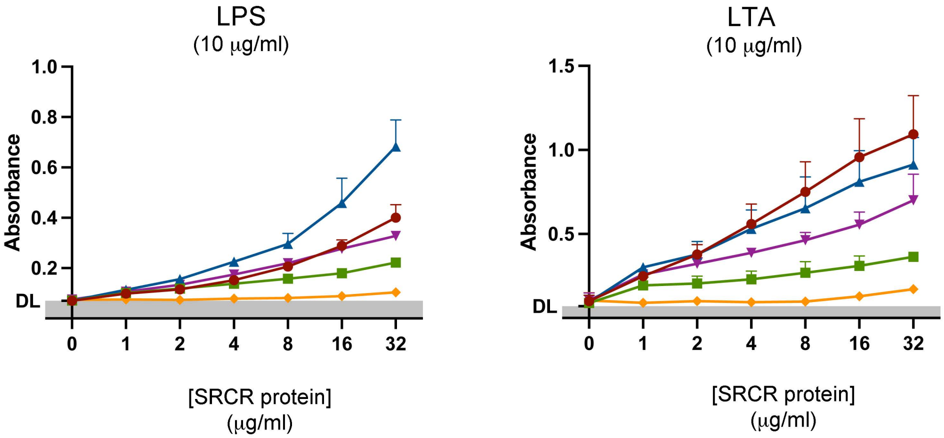

Supplemental Figure 3. Standard curve for detection of human SRCR proteins to LPS and LTA. (A) Serial dilutions of recombinant proteins SSC4D-FL, SSC4D-d1d2, SSC4D-d3d4, CD5L, and sCD6 were performed and proteins were directly coated in a 96-well plate. Plate-bound proteins were detected by anti-HIS antibody followed by HRP-conjugated antibody. Each point represents the mean ± SD of two independent experiments performed in duplicate. **(B)** 10 μg/ml of purified LPS or LTA were immobilized in a 96-well plate and incubated with different concentrations of recombinant SSC4D-FL, SSC4D-d1d2, SSC4D-d3d4, CD5L, or sCD6. Bound SRCR proteins were detected using anti-HIS antibody followed by HRP-conjugated antibody. Each point represents the mean ± SD of two experiments performed in duplicate.

Supplementary Figure 4

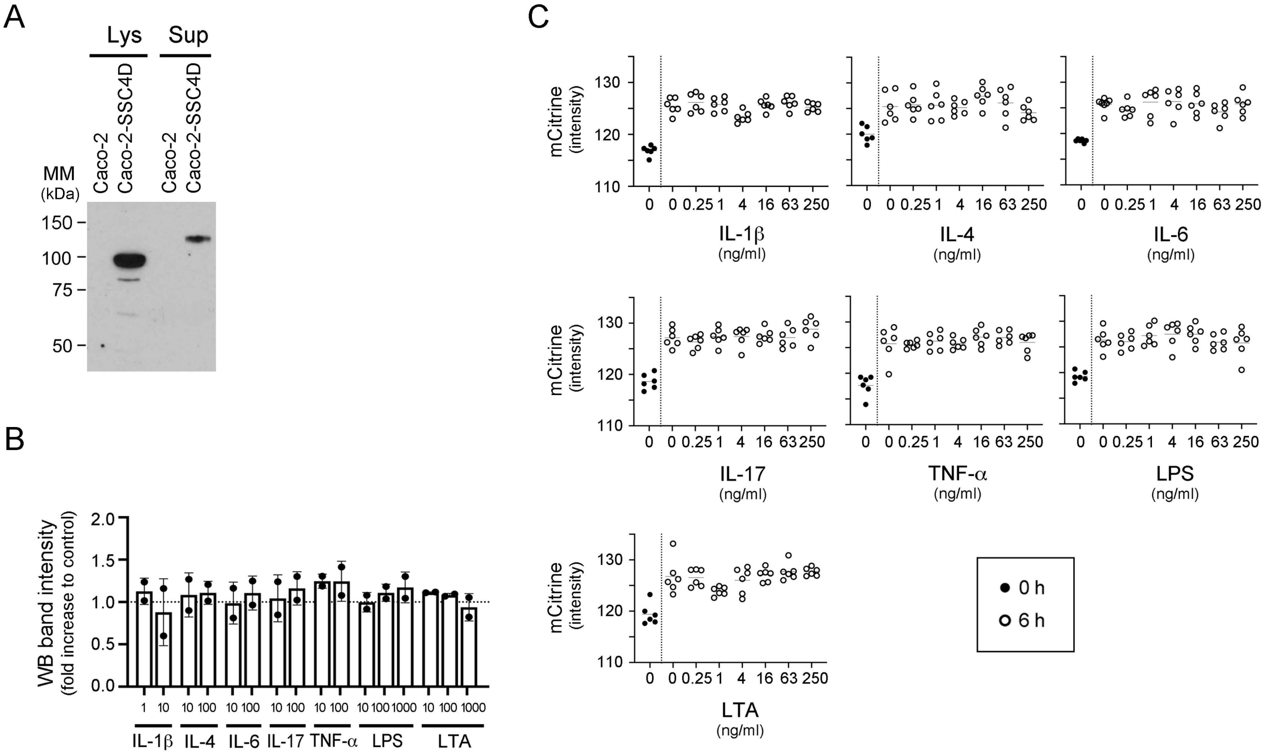

Supplemental Figure 4. SSC4D is secreted at constant rates, independently of external stimuli or cues. (A) Western blot of SSC4D fused to mCitrine and HA tag. The expressed protein displays a molecular mass of ~100 KDa when collected from cell lysates, compatible with the sum of the mass of SSC4D with those of mCitrine and HA, and of ~125 KDa as a secreted protein, also compatible with post-translational modifications observed earlier for recombinant SSC4D. **(B)** SSC4D secretion by Caco-2-SSC4D cells incubated for 24 h in the presence or absence of different stimuli (IL-1β, IL-4, IL-6, IL-17, TNF-α, LPS, and LTA) at different concentrations. The presence of SSC4D in the supernatants was analyzed by WB. Band densities were quantified and the values were normalized to the control (without stimulation). Graph shows the mean ± SD of two independent experiments. **(C)** Intracellular accumulation of SSC4D by Caco-2-SSC4D cells incubated for 6 h in the presence or absence of different stimuli (IL-1β, IL-4, IL-6, IL-17, TNF-α, LPS, and LTA) at different concentrations. Brefeldin A was used to block protein secretion, allowing for the determination of accumulation of intracellular SSC4D. Levels of intracellular SSC4D-mCitrine were analyzed using IN Cell. mCitrine intensity was measured before the addition of stimuli (0 h) and 6 h after the incubation with the different cytokines or endotoxins. In each time-point, 6 fields of view were analyzed for each well. Graphs shows one representative experiment of four with matching results, where mCitrine intensity values for each of the 6 fields of view were measured.
